# Myocardium T1 measurement using single and multi-shot SMART1Map acquisition: pros and cons

**DOI:** 10.1186/1532-429X-16-S1-P69

**Published:** 2014-01-16

**Authors:** Pauline Ferry, Anne Menini, Glenn S Slavin, Jeff A Stainsby, Damien Mandry, Laurent Bonnemains, Jacques Felblinger, Marine Beaumont

**Affiliations:** 1 IADI, Lorraine University, Vandoeuvre les Nancy, France; 2 U947, Inserm, Nancy, France; 3 GE Healthcare, Bethesda, Maryland, United States; 4 GE Healthcare, Toronto, Ontario, Canada; 5 CHU, Nancy, France; 6 CIT 801, Inserm, Nancy, France; 7 CIC-IT, CHU, Nancy, France

## Background

The recently published method SMART1Map[1] has proposed a new true T1 measurement technique. It consists in a 2D saturation-recovery prepared balanced-SSFP sequence which allows different acquisition schemes depending on the number of repetition (shot) used. Single-shot acquisition duration is short but cardiac motion blur can occur due to long acquisition window duration. Two-shot acquisition time allows an acquisition window twice smaller but inter acquisition window motion can occur. Note that single-shot scheme allows to acquire an additional point when magnetization has not yet undergo any saturation pulse, thus corresponding to an infinite saturation delay time (T∞). In this study, we compared both schemes on healthy volunteers to determine the most appropriate strategy for a large range of T1 values measurement at 3T.

## Methods

Our study was carried out in 6 volunteers on a 3T MR scanner (GE, Signa HDxt) using SMART1Map on one mid-cavity short-axis, in end-diastole, in breath-hold. Single-shot (matrix = 128*224) and two-shot (matrix = 160*224) strategies leading to 5 time points ranging from 50 ms to 2966 ms ± 473 ms were performed. We considered three data-sets: single-shot, two-shot and two-shot plus T∞ from single-shot. Breath-hold duration, chance of inter and/or intra acquisition motions, and spatial resolution were assessed for the different acquisition schemes. Post-processing T∞ was annotated with 9900 ms for fitting. For each data-set, pixel-wise T1 maps were generated. Data were fitted using a 3-parameter model. Due to sequence scheme, MR signal is likely to follow theoretical equation S(t) = M0-M0(1-cosθ)exp(-t/T1), usually modeled as S(t) = A-B*exp(-t/T1). On each T1 map, only the septum was encompassed for analysis, to overcome susceptibility or B1 effects on the lateral wall. For each ROI, average T1 with standard deviation were assessed. Data-set's mean T1 values with standard error of the mean (n = 6) were calculated; mean T1 standard deviations were also reported.

## Results

**Figure 1:**
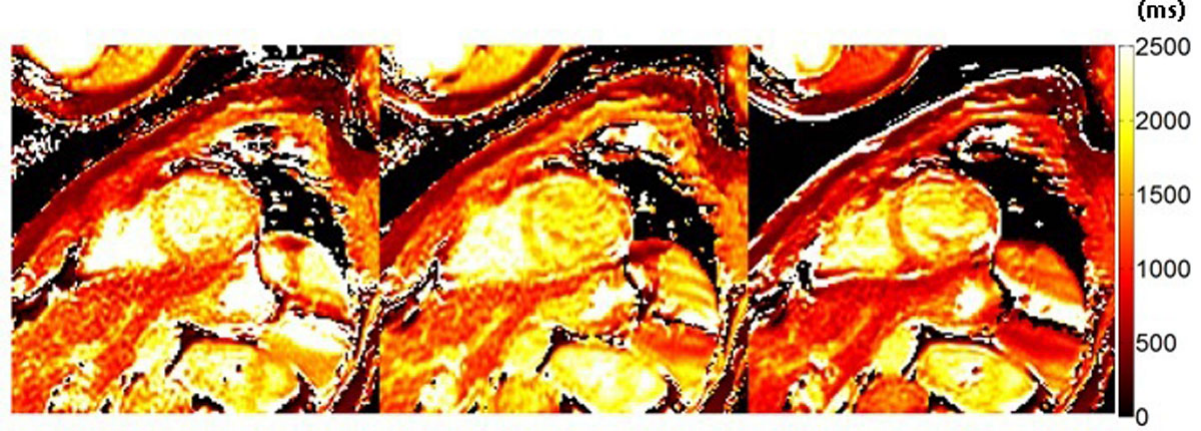
**T1 maps**. a, two-shot acquisition scheme. b, single-shot acquisition scheme. c, two-shot plus T∞ scheme.

**Figure 2:**
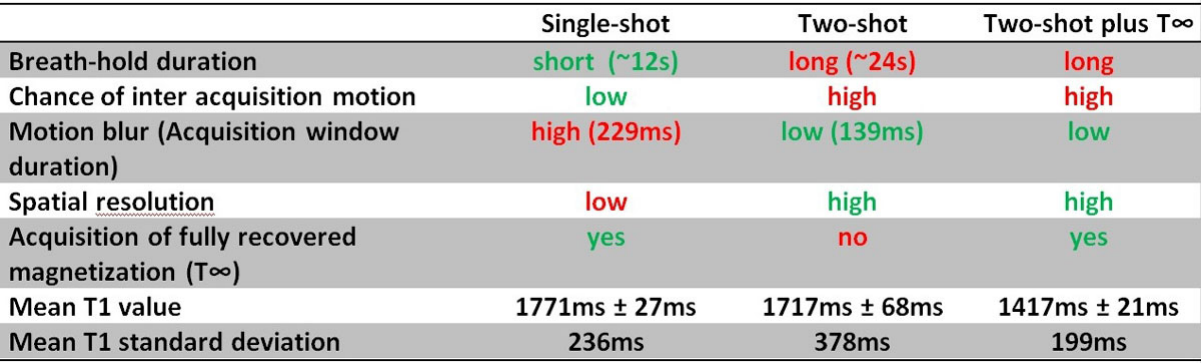
**Sequences characteristics**.

Myocardium is better defined on the two-shot plus T∞ data-set compared to others (Figure 1). Mean T1 value decreases when adding the T∞ to the two-shot data-set (Figure 2).

## Conclusions

Due to its long acquisition window, single-shot might lead to blurring limiting for focal T1 assessment. With a shorter acquisition window, two-shot acquisition is potentially more reliable, particularly when heart rate increases (shorter diastole period). However it suffers from poorly sampled recovery curves leading to noisy T1 maps. Adding T∞, from single-shot acquisition, to two-shot data-set allowed to stabilize fitting and resulted in better agreement with previous studies (myocardium T1 range: from 1471 ± 31 ms[2] to 1501 ± 69 ms[3]).

## Funding

FEDER, Région Lorraine, France.
